# Optimizing the feeding frequency to maximize the production of sterile males in tsetse mass-rearing colonies

**DOI:** 10.1371/journal.pone.0245503

**Published:** 2021-01-14

**Authors:** Soumaïla Pagabeleguem, Ange Irénée Toé, Sié Hermann Pooda, Kiswendsida Mikhailou Dera, Abdou Salam Belem, Adrien Marie Gaston Belem, Gisèle Marie Sophie Ouedraogo/Sanou, Mamadou Ira, Bénéwendé Aristide Kaboré, Lassané Percoma, Issa Sidibé

**Affiliations:** 1 Université de Dédougou (UDDG), Dédougou, Burkina Faso; 2 Insectarium de Bobo-Dioulasso - Campagne d’Eradication de la mouche Tsé-tsé et de la Trypanosomiase (IBD-CETT), Bobo-Dioulasso, Burkina Faso; 3 Ecole Privée d’Elevage et de Santé Animale (EPESA), Bobo-Dioulasso, Burkina Faso; 4 Université Nazi Boni (UNB), Bobo-Dioulasso, Burkina Faso; 5 Ecole de Lutte Anti Tsé-tsé (ELAT), Bobo-Dioulasso, Burkina Faso; 6 Centre International de Recherche-Développement sur l’Élevage en Zone Subhumide (CIRDES), Bobo-Dioulasso, Burkina Faso; Al-Azhar University, EGYPT

## Abstract

Tsetse flies are cyclical vectors of trypanosomes, the causative agents of sleeping sickness or Human African Trypanosomosis and nagana or African Animal Trypanosomosis in Sub-Saharan Africa. The Insectarium de Bobo-Dioulasso (IBD) was created and equipped in the frame of Pan African Tsetse and Trypanosomosis Eradication Campaign (PATTEC) with the main goal to provide sterile males for the different eradication programs in West Africa which is already the case with the ongoing eradication program in Senegal. The aim of this study was to identify the best feeding regime in mass-rearing colonies of *Glossina palpalis gambiensis* to optimize the yield of sterile males. We investigated the mortality and fecundity for various feeding regimes and day alternation (3×: Monday-Wednesday-Friday, 4×: Monday-Wednesday-Friday-Saturday, 4×: Monday-Wednesday-Thursday-Friday and 6×: all days except Sunday) on adult tsetse flies in routine rearing over 60 days after emergence. The day alternation in the 4 blood meals per week (feeding regimes 2 and 3) had no effect on tsetse fly mortality and fecundity. The best feeding regime was the regime of 4 blood meals per week which resulted in higher significant fecundity (PPIF = 2.5; P = 0.003) combined with lower mortality of females (P = 0.0003) than the 3 blood meals per week (PPIF = 2.0) and in similar fecundity (PPIF = 2.6; P = 0.70) and mortality (P = 0.51) than the 6 blood meals per week. This feeding regime was extended to the whole colonies, resulting in an improved yield of sterile males for the ongoing eradication program in Senegal and would be more cost-effective for the implementation of the next-coming sterile insect technique (SIT) programs in West Africa.

## Background

Tsetse flies transmit trypanosomes which cause sleeping sickness or Human African Trypanosomoses (HAT) and nagana or African Animal Trypanosomoses (AAT), a debilitating diseases affecting humans and livestock, respectively [1]. The tsetse flies infest 38 countries of sub-Saharan Africa constraining the development of sustainable and productive agricultural systems in over 10 million km^2^ [2]. The presence of this insect leads to potential losses in livestock and crop production estimated at USD 4,750 million annually [3]. In this context, vector control is considered as an important component of the integrated management of both HAT and AAT. The control of these diseases within an area-wide integrated pest management approach (AW-IPM) using the sterile insect technique (SIT) has shown its effectiveness in Burkina Faso [4], Nigeria [5] and Zanzibar [6]. Motivated by these encouraging results, the African Heads of States and Governments committed for better food security through better management of the tsetse flies and the parasites they transmit by creating in 2000 in Lome the Pan African Tsetse and Trypanosomosis Eradication Campaign (PATTEC) [7]. In Burkina Faso, 83% reduction of densities was observed for *Glossina palpalis gambiensis* and 92% reduction for *G*. *tachinoides* on 40000 km^2^ has been achieved through an integrated control campaign including insecticide targets, traps and cattle, sequential aerial treatment (SAT) and the mass treatment of livestock using trypanocidal products, from June 2006 to December 2013 [8]. Moreover, during this phase of PATTEC Burkina, a mass-rearing facility named Insectarium de Bobo-Dioulasso (IBD) was built and equipped with capacities to produce about 1,000,000 sterile males weekly in cruise production. The main goal of this facility is to satisfy the needs in sterile males for the program of Burkina Faso and the other countries infected by the same tsetse species. The rearing started in July 2016 and currently, two species of flies are mass-reared, *G*. *palpalis gambiensis* and *G*. *morsitans submorsitans*. Some initiatives are underway to establish *G*. *tachinoides* colony.

Under the PATTEC initiative, the Government of Senegal initiated in 2005 a tsetse flies eradication program in the Niayes area that integrated the SIT with other control tactics such as insecticides treated targets and insecticides treated cattle [9–11]. For the SIT component, an agreement was made with the IBD to mass-produce *G*. *p*. *gambiensis* to supply 50,000 sterile male pupae weekly to Senegal for this AW-IPM program since 2017. The tsetse flies colony should double every 3–4 months if all conditions are optimal [12]. Since the start of this agreement, the IBD has not been able to supply the necessary quantity of sterile pupae, despite the improving of rearing conditions, thus limiting the possibilities of complying with the agreement.

In order to respond to that demand for the ongoing program in Senegal and the near future programs in Africa, IBD has recently undertaken experiments aiming to improve the rearing system and the quality of the tsetse flies produced. Indeed, *In vitro* blood feeding, the major factor determining the productivity and survival rate of tsetse, Glossina species is the qualitative and quantitative amount of blood imbibed during the interlarval period [13]. This study was therefore initiated to test various feeding regime to optimize the production of sterile males in the IBD tsetse mass-rearing facility.

## Materials and methods

### Insectary

The study was carried out at the Insectarium de Bobo-Dioulasso (IBD) localized at Darsalamy, 15 Km from Bobo-Dioulasso (11°03’32.4"N and 4°21’10.9"W), Burkina Faso. The rearing rooms included air circulation, cooling and humidification to manage environmental parameters. The temperature and relative humidity of the holding rooms were kept at a standard tsetse colony rearing conditions 25 ± 1°C, 75 ± 5% RH [14] during the tests (see supplementary material (S1 Fig) for recorded data) and 12:12 light:dark photoperiod for pupae incubation, emergence, feeding and the monitoring of the flies.

### Tsetse species and strain

All experiments were carried out with a strain of *G*. *p*. *gambiensis* fed on silicone membranes at the IBD. The colony was derived from a strain that was established at Maisons-Alfort, France in 1972 and originated from Guinguette, a locality near Bobo-Dioulasso, Burkina Faso. It was transferred in 1975 to the Centre de Recherche sur les Trypanosomiases Animales (CRTA), Burkina Faso [15–17] [CRTA is the former name of the Centre International de Recherche-Développement sur l’Elevage en zone Subhumide (CIRDES)]. In 2016, 53,972 adult flies of this CIRDES colony were transferred to the IBD to where a colony was likewise established for mass production.

### Blood origin and preparation

The bovine blood used to feed the *G*. *p*. *gambiensis* colony was collected from the slaughterhouse of Bobo-Dioulasso, with the consent from the slaughterhouse managers in order to obtain the blood samples from livestock. The blood was immediately defibrinated after collection and transported to laboratory for proportioning and quality control operations. Previously frozen at -20°C, the blood was irradiated with 1 KGry in a Cobalt 60 irradiator (model 812, Sn 002). The blood quality-control was tested after irradiation through the microbial contamination value that consisted of bacteriological cultures on agarose middle in petri dishes at 37°C. After 72 hours incubation, the blood batch is declared of good quality if the number of microbial colonies is less than 10 and used to feed the tsetse colony. Aliquots of the blood are thawed and used as required. The experimental flies were fed on an *in vitro* silicone membrane system with the blood which was heated to 36–37°C on controlled aluminum plates of Tsetse Production Unit (TPU4).

### Feeding regime

The study was run from March to May, 2018 and included 480 *G*. *p*. *gambiensis* (360 females and 120 males) from the same production batch (Table 1) and follow up at the same period of time.

**Table 1:** Number of females and male *Glossina palpalis gambienis* tested according to the feeding regimes.

Feeding regime/week	Males	Females	Number of cages
1 (3×: mon-wed-fri)	30	90	3
2 (4×: mon-wed-fri-sat)	30	90	3
3 (4×: mon-wed-thu-fri)	30	90	3
4 (6×: all days except Sunday)	30	90	3

mon = Monday, wed = Wednesday, fri = Friday, thu = Thursday, sat = Saturday.

Newly emerged flies were separated by sex and put into cages (13cm x 5cm x 8cm) covered with white tulle of large mesh (2.5 mm) following the initial male to female ratio of 1:3 [18]. The large mesh allowed third instar larvae (L3) to escape from the cage. The cages were placed in the same room as the main colony. Males and females remained together until the end of the experiment.

Four feeding regimes were defined and differed from the number of blood meals per week as well as the alternation of days: regime 1: 3 times (Monday-Wednesday-Friday), regime 2: 4 times (Monday-Wednesday-Friday-Saturday), regime 3: 4 times (Monday-Wednesday-Thursday-Friday) and regime 4: 6 times (all days except Sunday). Those alternations of days had been chosen according to the work plan in the insectary, in order to be able to apply the best treatment with the technicians. Three cages by treatment were set and each cage contained 30 females and 10 males (Table 1).

### Fecundity and reproductive biology

Mating cages were placed in individual larviposition cups and pupae were collected daily (except Sundays) and sorted into normal and aborted L3. The normal pupae were weighed using an electronic balance of 0.0001 mg sensitivity and automatic calibration (Sartorius MSE2 7S-000-DM Cubis Ultra). The production of pupae was recorded daily per feeding regime and per cage. The first larval period (time between female emergence and the production of the first pupae) and the pupae produced per initial female (PPIF) was also recorded.

### Male and female adult mortality

Mortality was recorded daily (except on Sundays) for each treatment and per cage until 60 days after emergence. Dead flies were sorted into blood-fed and starved fly mortalities.

### Flight ability assessment in the insectarium

In the framework of the supplying in the sterile males to the ongoing AW-IPM program in Senegal, a standard quality control protocol was applied at the IBD. Flight ability data of *G*. *p*. *gambiensis* colony fed 4 times per week were collected from March to November 2019. Sterile males pupae are shipped twice per week to Senegal and at each shipment, 100 pupae were put in Petri dishes covered by a flight cylinder, i.e. a PVC tube 10 cm high and 8.4 cm in diameter [19,20]. The inner wall of the cylinder was coated with unscented talcum powder to prevent the flies from crawling out. Flies flying out of the tube were considered as “operational flies” (i.e. available for the SIT) [19,20]. This control protocol is to evaluate the quality of sterile males that is a quality indicator of the IBD *G*. *p*. *gambiensis* colony.

### Statistical analysis

The R Software (version 4.0.3) [21] using RStudio (RStudio, PBC. Boston, MA, USA, 2020) was used to perform the statistical analyses and the figures. The Shapiro-Wilk test was used to test the normality of data and the Tukey’s test was applied. If the data was not normally distributed the nonparametric Kruskal-Wallis test was used then transformed to be normally distributed using the transformTukey function in the *rcompanion* package [22]. The survival of flies was analyzed using Kaplan-Meier survival curves and were compared using the coxme model [23] where the cage number was considered as a random effect, the feeding regime, the sex and their second order interactions were used as explanatory variables and survival rate as the response variable. The significant interactions were analyzed using the emmeans function (in package *emmeans*) [24]. A Generalized linear mixed effect model was used to analyze the number of pupae produced per initial female (PPIF) (with a Poisson distribution) and the pupal mass after 60 days of emergence (with a Gaussian distribution) [25] where the feeding regime, the cage and their interactions were considered as fixed variables. Differences between the levels of significant fixed factors were analyzed using post hoc Tukey tests (glht function in package *multcomp*) [26]. All tests were done at the 5% significance level. The data analysis details are available as supplementary file (S1 Data).

### Data accessibility

The complete data sets are available in S1–S4 Tables.

## Results

### Mortality

Overall dead flies (males and females), 2.82% had blood in the abdomen and 97.17% had starved. The statistical analysis showed that there was no link between the number of deaths with or without blood in the insect’s abdomen and the feeding regime and sex (P > 0.05).

**Fig 1:**
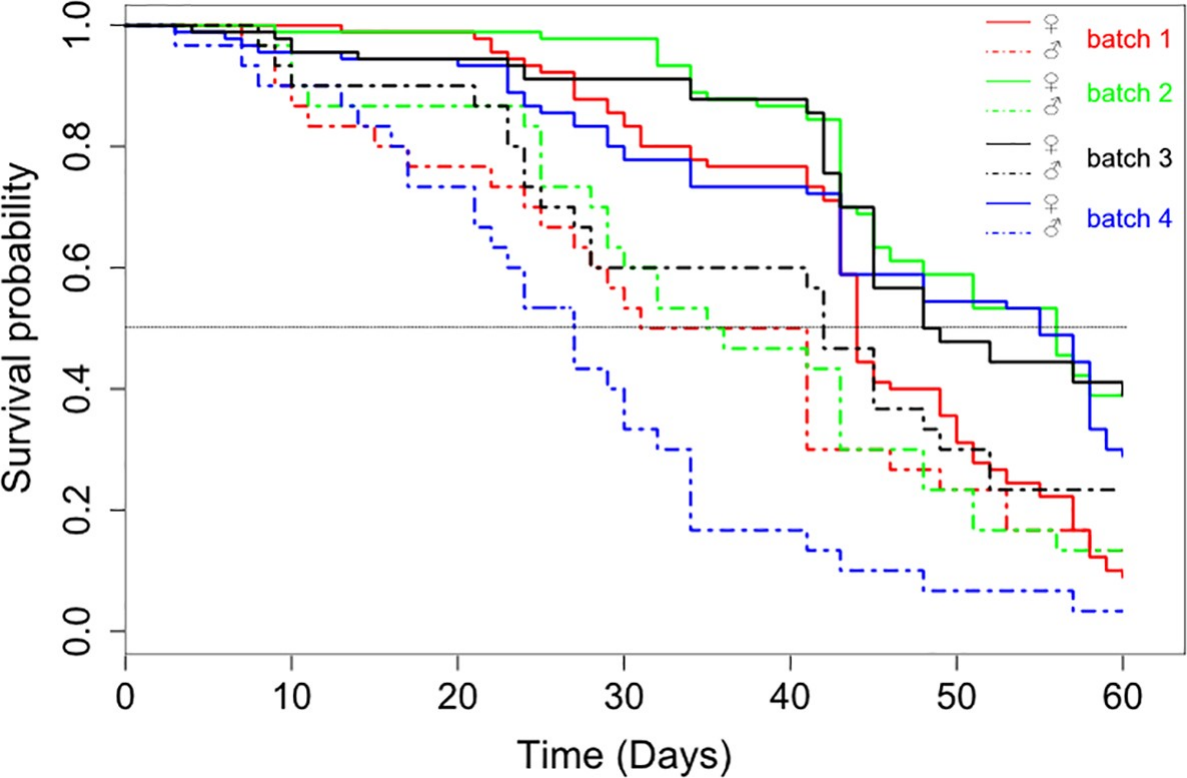
Survival curves of flies maintained on different feeding regimes and by sex.

The mortality rate was significantly influenced by the feeding regime (X^**2**^_**3**_ = 20. 87, P < 0.001, Fig 1) and the sex (X^**2**^_**1**_ = 43.95, P < 0.001, Fig 1). Moreover, the interaction between sex and feeding regime was significant (X^**2**^_**1**_ = 15.76, P = 0.001), showing that the effect of the feeding regime was not the same on the survival of females and males. Indeed, females survived significantly longer than males, irrespective of the feeding regime (P < 0.001; Table 2). For the females, the comparison between feeding regimes showed that the mortality rate was similar between flies fed 4 times a week (feeding regimes 2 and 3) and flies fed 6 times a week (feeding regime 4) (P > 0.2). While, the mortality rate was significantly higher in the feeding regime 1 (3 blood meals by week) compared to 3 other feeding regimes (P < 0.002; Table 2). For the males, the survival was similar between the feeding regimes (P > 0.1; Table 2). The day alternation in the 4 blood meals per week had no effect on tsetse fly mortality.

**Table 2:** Summary of the best cox model for the survival of flies per sex after 60 days.

Fixed effect	coef	exp(coef)	se(coef)	z-value	Pr(>|z|)
Females					
feeding regime 2	-0.7788	0.4589	0.2119	-3.68	0.0002***
feeding regime 3	-0.7020	0.4956	0.2121	-3.31	0.0009***
feeding regime 4	-0.5234	0.5925	0.2061	-2.54	0.011*
Males					
feeding regime 2	-0.0622	0.9397	0.3771	-0.17	0.870
feeding regime 3	-0.3085	0.7346	0.3834	-0.80	0.420
feeding regime 4	0.6188	1.8567	0.3743	1.65	0.098.

The feeding regime 1 (3 blood meals per week) is here considered as the reference level.

Abbreviation: Coef, coefficient; SE, standard error.

Significance:

***P ≤ 0.001;

* P ≤ 0.05 (these apply to values above).

### Productivity

**Fig 2:**
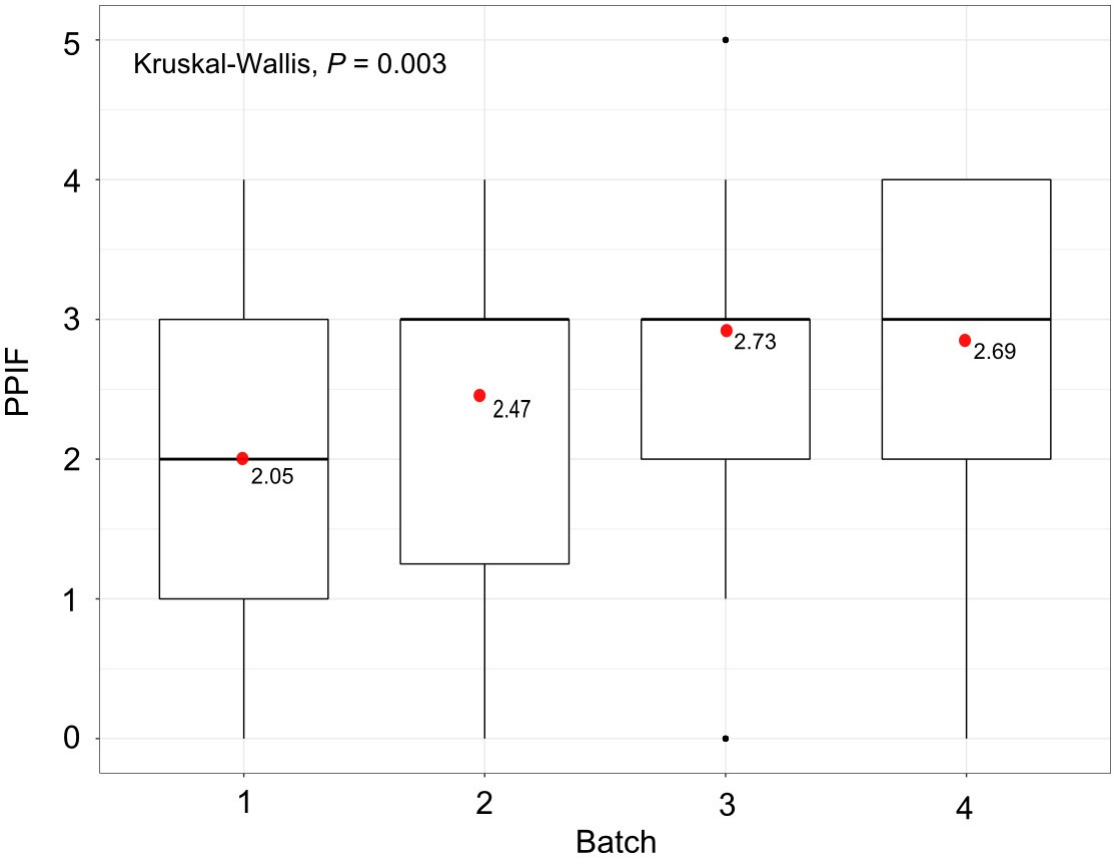
Number of pupae produced per initial female (PPIF) after 60 days according to the number of blood meals per week and the alternation of the days. The red points give the mean PPIF for each batch.

The first larva was deposited on average on day 20 for the feeding regimes 2, 3 and 4 (4 and 6 blood meals by week) and 24 hours after for the feeding regime 1 (day 21) (3 meals by week). The results indicated that at 60 days, the highest fecundity was observed for the feeding regime 3 (4 blood meals by week) (2.73 ± 1.32 PPIF). However, this did not differ significantly from the feeding regimes 2 (2.47 ± 1.31) and 4 (2.69 ± 1.28) (fed 4 and 6 times by week respectively, P > 0.3; Table 3). The lowest fecundity was observed for the feeding regime 1 (3 blood meals by week) (2.05 ± 1.36 PPIF) that was significantly different from the three others (P < 0.04; Fig 2; Table 3). The pupal mass was statistically similar between feeding regimes 1, 2 and 3 (P > 0.5; Table 3) and significantly different to that of the feeding regime 4 (P < 0.03); mean (± SD) values of 24.00 ± 2.01, 23.85 ± 2.29, 23.97 ± 2.10, 24.10 ± 0.20 and 24.56 ± 2.08 mg were obtained for the feeding regimes 1, 2, 3 and 4, respectively.

**Table 3:** Fixed-effects coefficients of a mixed-effect Gaussian model of the impact of the number of blood meals by week and the alternation of days on the number of pupae produced per initial female (PPIF) and pupal mass after 60 days.

Trait	Fixed effect	Estimate	Standard Error	z-value	Pr(>|z|)
PPIF	(Intercerpt)	1.0573	0.0621	17.018	< 2e-16***
	feeding regime 2	0.2083	0.0836	2.491	0.0128*
	feeding regime 3	0.3338	0.0814	4.101	4.11e-05***
	feeding regime 4	0.3089	0.0818	3.776	0.00016***
Pupal mass	(Intercerpt)	222.9581	2.4728	90.166	<2e-16***
	feeding regime 2	-2.1771	3.3667	-0.647	0.5180
	feeding regime 3	-0.3749	3.2692	-0.115	0.9087
	feeding regime 4	8.8992	3.2715	2.715	0.007**

The feeding regime 1 (3 blood meals per week) is here considered as the reference level.

Significance:

***P ≤ 0.001;

** P ≤ 0.01;

* P ≤ 0.05 (these apply to values above).

### Flight ability in the insectarium

**Fig 3:**
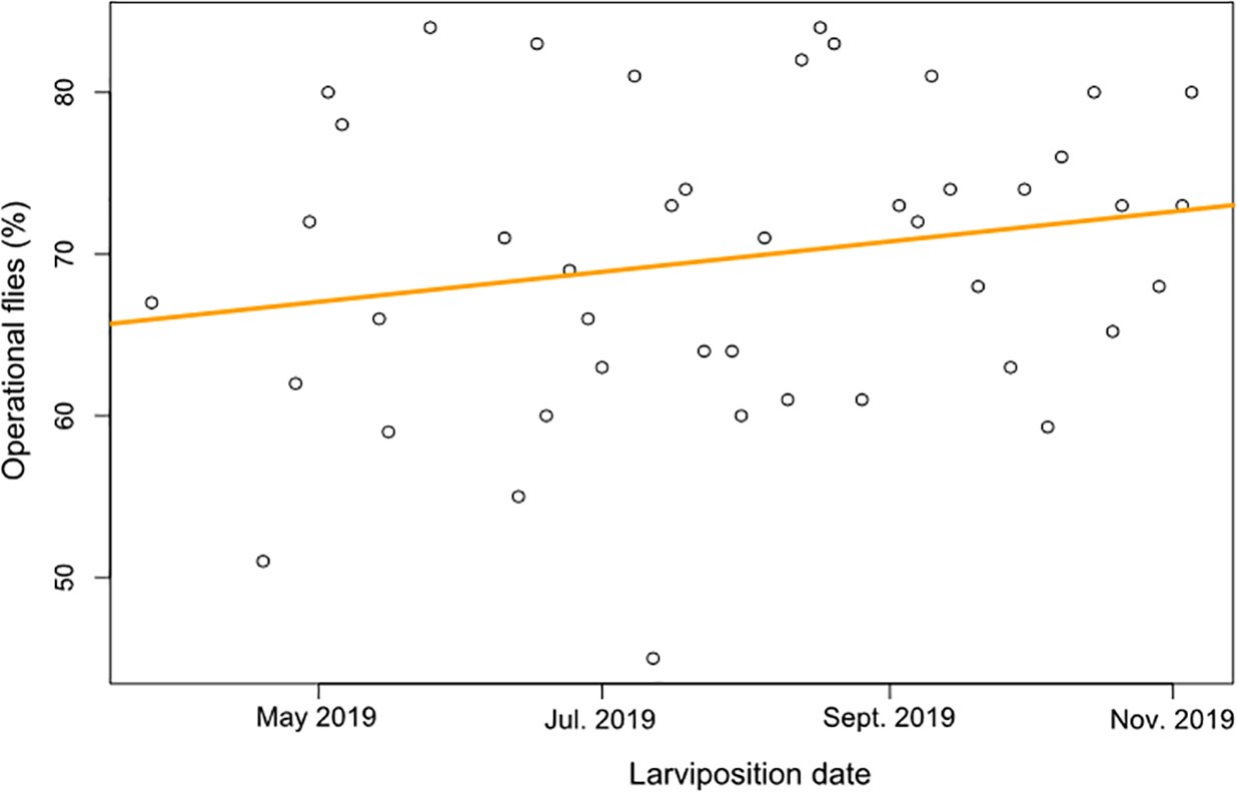
Percentages of operational flies (%) over the time. The orange line gives the trend of performance evolution over the time.

The flight ability data of 4378 pupae were recorded from March to November 2019 after the passing from 3 to 4 blood meals per week to the full colonies. The proportion of operational flies was increased with the time corresponding to an average of 6% improving of the quality of IBD colony (Fig 3).

## Discussion

The aim of this study was to assess the survival and productivity of adult *G*. *p*. *gambiensis* flies in colony cages at different feeding regimes per week and at alternation of the days.

We observed a low proportion (2.82%) of dead flies with blood in the abdomen. This means that the blood used to feed the experimental flies was of good quality and did not cause the observed mortalities.

We found that female flies survived significantly longer than males irrespective of the treatment. The similar results were obtained in previous studies under temperature variations with the same species, *G*. *p*. *gambiensis* [27] and with mosquitoes, such as *Anopheles arabiensis*, *An*. *funestus* [28] and *Aedes albopictus* [29]. The difference in lifespan between the sexes is common in insects and it seems to be genetically determined [30].

The results showed that under 25°C, the survival and productivity of flies that had 3 blood meals per week were lower than those fed 4 or 6 times per week. Our results are in line with the previously obtained data on *G*. *pallidipes* and *G*. *morsitans morsitans* [31]. This previous study on feeding regime in relation to reproduction showed a positive correlation between feeding regime and adult survival and fecundity. Indeed, a minimum number of blood meals per cycle (an inter-larval period of 9 days) which did not cause a decline in reproductive performance was 4 times for *G*. *m*. *morsitans* and five for *G*. *pallidipes* [31]. According to Gaston and Randolph (1993), the flies fed every third day, always engorged fully at every opportunity, whereas, flies offered food every second day refused to feed at all or did not engorge fully at every opportunity [32]. Our results confirm the previous study showing that in the laboratory, female flies which take a full blood meal every third day perform less (survival and productivity) than those which take on average rather smaller blood meals every second day [31,32]. Indeed, experiments carries out on *G*. *morsitans* showed that the greater proportion of larger meals is taken on day 7 of the inter-laval period [33,34] corresponding to the time of rapid larval growth [32]. This meal that is critical for normal larval development, could explain the better productivity observed with flies fed 4 times per week than those fed 3 times, since with the 4 meals, the flies have more opportunity to have meal at the critical time.

In other tsetse facilities in Europe as at the FAO/IAEA Insect Pest Control Laboratory (IPCL) in Seibersdorf, Austria and the Slovak Academy of Sciences (SAS) in Bratislava, Slovakia where the same species *G*. *p*. *gambiensis* is reared but not massively as that of IBD, 3 days feeding per week is used with acceptable production [12]. However, the using of the same 3 days feeding per week for the same tsetse species at the IBD and at the CIRDES facilities in Bobo-Dioulasso, Burkina Faso [35] and for *G*. *pallidipes* at the Ethiopian Tsetse Eradication Project (STEP) facility [36], doesn’t allow to reach the objective of production. Indeed, a reduction of the feeding frequency from 6 to 4 times per week for the CIRDES mass rearing colony of *G*. *p*. *gambiensis* [35] and an increasing from 3 to 5 meals per pregnancy cycle of the STEP mass rearing colony of *G*. *pallidipes* [36] had been demonstrated. Our results are in agreement with the findings by these authors who observed also that 3 blood meals per week affecting the mass rearing production (high mortality and low fecundity). Indeed, the quality of the blood used for the feeding could be the difference between European and African insectariums. The waiting period of veterinary drugs before the slaughter of the animal is well respected in Europe, which is not the same everywhere in Africa, so some drug residues could end up in the blood and impact the quality of the production, despite the biological feeding test that eliminates very poor quality blood (Quality Factor <1, [12,37]).

Following our results, 4 blood meals per week (Monday, Tuesday, Thursday and Friday) was applied to the full colonies at IBD (Instead of 3 meals per week, Monday, Wednesday and Friday) since December 2018, reducing then female mortality, increasing colony productivity and size. However, the young flies (less than one week old) are fed additionally on Wednesday and Saturday. As a reminder, in the framework of the ongoing elimination program in Senegal, an agreement was made with the IBD to mass-produce *G*. *p*. *gambiensis* to supply the sterile males needed for the SIT component of this AW-IPM program. Considering the *G*. *p*. *gambiensis* colony, the main target for this program, the size constituted of ~ 617,600 females on week 42 of 2019, corresponding to a weekly pupae collection of 130,400. Thus, the change of the feeding regime allowed increasing the number of sterile male pupae supplied to Senegal project from less than 30,000 per week in December 2018 to more than 50,000 in June 2019 and contributed to reach the projected objective of receiving at ~ 50,000 sterile male pupae weekly.

Three to 4 meals per week would increase the quantity of blood used per week leading to an increase of the workload for the insectarium technical workers including blood collection at the slaughterhouse and the feeding activities. Indeed, with 617,000 females as colony size, around 50 liters of blood are used at each feeding. The cost of defibrinated and irradiated blood is less than 2 Euros per liter, i.e. a total cost of less than 80 Euros per day. From 30,000 sterile male pupae supplied to Senegal project per week in December 2018 to more than 50,000 in June 2019, the 20,000 pupae increase brings in 3,000 euros per week, without forgetting the 50% of females that will be re-injected into the colony. About the manpower for a day feeding, it should be noted that the staff of the IBD is not paid by the hour, but by monthly lump sum, therefore the increase in the number of meals does not lead to an increase in personnel costs. The additional charges would be related to depreciation of equipment and energy costs. However, an economic study will be necessary to verify the cost effectiveness of the production of sterile male flies according to the production systems in different facilities. For the performance of flies, a recent study carried out at CIRDES shown that the 4 meals per week gave a better flight ability than 3 meals per week [35]. Which supports the performance that we obtained with the IBD colony after the application of 4 meals per week, which has gradually improved over time, from 67% in March 2019 to 76.5% in November 2019 using pupae subjected to chilling (8 ± 2°C) for 24 to 72 hours followed by an irradiation treatment.

The number of blood meals per week was increased to 4 only and not 6 which gave similar results. Since 6 blood meals per week gave a similar survival and productivity, using a target 4 blood meals per week is thus more conservative.

## Conclusion

The best number of blood meals per week should be of 4 times for *G*. *p*. *gambiensis* to enhance its longevity and productivity. Thus, this mass-production improvement would allow the IBD to meet the needs for sterile males for the ongoing eradication program in Senegal and would give more capacity for the implementation of the future SIT programs in West Africa, such as in Burkina Faso and Chad.

## Supporting information

S1 Fig (TIF)

S1 Table Database for the survival of flies maintained on different feeding regimes. (CSV)

S2 Table Database for the pupae produced per initial female after 60 days. (CSV)

S3 Table Database for the pupal mass after 60 days according to the number of blood meals by week. (CSV)

S4 Table Database for the flight ability assessment in the insectarium. (CSV)

S1 Data (RMD)
