# Supplementary figures and images for: Optimizing the feeding frequency to maximize the production of sterile males in tsetse mass-rearing colonies
Source: PLoS One. 2021 Jan 14;16(1):e0245503. doi: 10.1371/journal.pone.0245503 (PMC7808581; doi:10.1371/journal.pone.0245503)

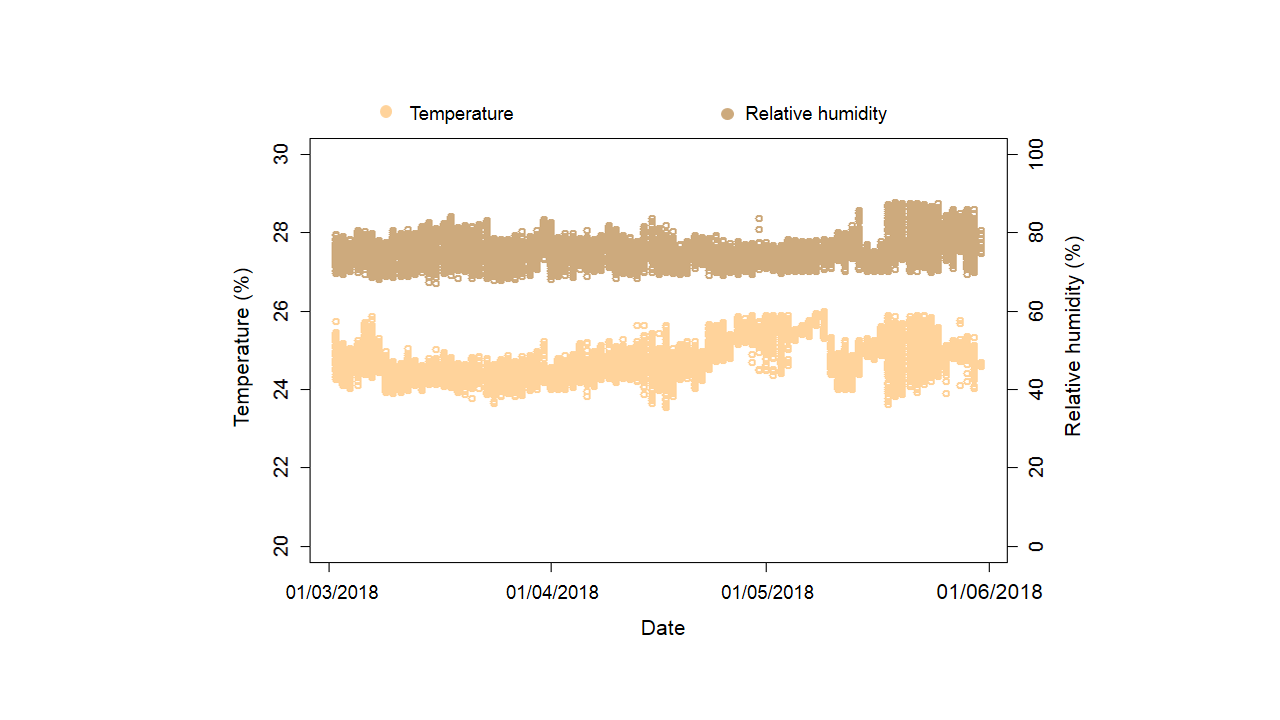

Supplement: S1 Fig — (TIF) [file pone.0245503.s001.tif]
